# Psychological factors outmatched morphological markers in predicting limitations in activities of daily living and participation in patients with lumbar stenosis

**DOI:** 10.1186/s12891-019-2918-0

**Published:** 2019-11-23

**Authors:** V. Quack, M. Boecker, C. A. Mueller, V. Mainz, M. Geiger, A. W. Heinemann, M. Betsch, Y. El Mansy

**Affiliations:** 1 Department of Orthopedic Surgery, RWTH Aachen University, Aachen, Germany; 2 Department of Neurosurgery, RWTH Aachen University, Aachen, Germany; 3 Department of Medical Psychology and Medical Sociology, RWTH Aachen University, Aachen, Germany; 4 Department of Physical Medicine and Rehabilitation, Feinberg School of Medicine, Northwestern University, Chicago, IL USA; 5 Center for Rehabilitation Outcomes Research, Shirley Ryan AbilityLab, Chicago, IL USA; 6 The Orthopedic Department, Alexandria University, Alexandria, Egypt

**Keywords:** Lumbar stenosis, Predictors, Morphological markers, Psychological factors, Depression, Fear of movement, Outcome

## Abstract

**Background:**

Recent demographic changes have led to a large population of older adults, many of whom experience degenerative disc diseases. Degenerative lumbar spinal stenosis (DLSS) is associated with considerable discomfort and limitations in activities of daily living (ADL). Symptomatic DLSS is one of the most frequent indications for spinal surgery. The aim of this study was to identify sociodemographic variables, morphological markers, depression as well as fear of movement that predict ADL performance and participation in social life in patients with DLSS.

**Methods:**

Sixty-seven patients with DLSS (mean age 62.5 years [11.7], 50.7% females) participated in the study. Predictor variables were age, gender, duration of disease, three morphological markers (severity of the lumbar stenosis, the number of affected segments and presence of spondylolisthesis) as well as self-reported depression and fear of movement. Dependent variables were pain interference with the performance of ADLs, ADLs and participation in social life. Correlations between predictor and dependent variables were calculated before stepwise, linear regression analyses. Only significant correlations were included in the linear regression analyses.

**Results:**

Variance explained by the predictor variables ranged between 12% (R^2^ = .12; pain interference-physical) and 40% (R^2^ = .40; ADL requiring lower extremity functioning; participation). Depression and fear of movement were the most powerful predictors for all dependent variables. Among the morphological markers only stenosis severity contributed to the prediction of ADLs requiring lower extremity functioning.

**Conclusion:**

Depression and fear of movement were more important predictors of the execution of ADLs and participation in social life compared to morphological markers. Elevated depressive symptoms and fear of movement might indicate limited adaptation and coping regarding the disease and its consequences. Early monitoring of these predictors should therefore be conducted in every spine centre. Future studies should investigate whether psychological screening or a preoperative psychological consultation helps to avoid operations and enables better patient outcomes.

## Background

Demographic changes in the last decades led to an increased number of elderly people going along with an increase of the prevalence of degenerative disc diseases. Degenerative lumbar spinal stenosis (DLSS) is related to narrowing of the lumbar spinal canal [1] and is associated with degenerative changes of the joint complex, osteophyte formation and ligamentum flavum thickening [2]. Degeneration begins at the intervertebral disc that may cause instability of the spine segment leading to a degenerative cascade of the spine unit [2]. The prevalence of this condition, based on radiographic criteria of patients older than 60 years, is estimated to be about 50% [3]. The clinical prevalence of adults with symptoms of pain and numbness referred to the lower extremities is about 47% [4]. Patients with DLSS may have several clinical symptoms including radiating leg pain, improvement of the pain when bending forward and gait disturbances. This often results in significant reductions in activities of daily living (ADL).

There is no “gold standard” for the diagnosis of DLSS [5]. Clinical judgement is generally based on symptom severity, functional deficits, physical examination and radiographic imaging [6]. The most promising imaging test is magnetic resonance imaging (MRI) [5]. Unfortunately, older adults often demonstrate abnormal MRI findings, even in those who are asymptomatic [7].

Only a few studies have investigated how MRI-based morphological markers are associated with symptom severity and functional deficits. Zeifang et al. found that the severity of lumbar stenosis (cross-sectional area of dural sac) and walking distance in gait analysis are not associated [8]. Likewise, Sigmundsson et al. reported a weak correlation between walking distance, pain interference with ADLs, quality of life and leg and back pain levels and the cross-sectional area of the dural sac. However, they found better general health and less leg and back pain in patients with multilevel stenosis than in patients with single-level stenosis, probably because of adaptation such that patients with a longer history of pain and limitations are more able to accept and handle the consequences of disease [9]. Kuittinen et al. compared the radiological evaluations of DLSS (classification in three severity classes based on MRI inspection of the dural sac area) with clinical findings (e.g., pain interference, pain intensity, depression, walking distance). They found no clear association between the severity of DLSS and clinical findings, but reported partly milder symptoms in patients with more severe stenosis [10]. In contrast, Hong et al. found a significant, albeit small correlation between the number of affected segments (single vs. multilevel DLSS) and pain interference [11]. Taken together, the evidence regarding the association between morphological markers and functional disability reveals small but inconsistent effects. Consequently, it remains to be elucidated which other factors influence functional disability in patients with DLSS.

It is long known that low back pain is associated with psychosocial impairment, poor sleep quality and depression [12]. Patients presenting with significant psychosocial distress at surgical evaluation have a lower quality of life and more severe physical disability. After surgery, patients with moderate psychosocial distress have poorer clinical outcomes, although, in comparison to the preoperative baseline, they improved [13]. In a 10-year follow-up after decompression surgery for lumbar stenosis, Tuomainen et al. reported that patients with lumbar stenosis with elevated depressive symptoms had an increased risk of postoperative pain and disability in the long term follow-up than patients without depressive symptoms [14].

Celestin et al. reported a systematic review of variables that predict pain-related treatment outcomes in the interventional treatment of chronic back pain. They concluded that psychological factors including somatization, depression, anxiety, and poor coping, are important predictors with greater risk of poor postoperative outcome [15]. Similarly, Lee reported associations between preoperative anxiety, optimism and postoperative patient satisfaction [16], and Ekman reported that patient factors including sex, age, preoperative working and exercise status influence postoperative outcomes [17]. Methodological factors like the use of different screening questionnaires, short follow-ups and different indications for spine surgery make a comparison of studies a challenge.

Continuing demographic changes, increased reliance on surgery for DLSS, the increasing use of MRI imaging for DLSS diagnosis, the weak association between radiographic findings and clinical symptoms, and the influence of psychosocial factors on treatment outcome make it critical to improve our understanding of this disease pattern. Thus, the aim of this study was to evaluate the relative influence of sociodemographic characteristics, morphological markers, fear of movement as well as the degree of depression with respect to the prediction of ADL in patients with DLSS.

## Methods

### Study population

Sixty-seven in- and outpatients diagnosed with DLSS participated in this study. They were treated in a neurosurgery clinic and an orthopedic surgery clinic between May 2015 and June 2016. The data were collected as part of a larger study with patients with diverse spinal diseases which aimed at developing an instrument assessing environmental factors based on the International Classification of Functioning, Disability and Health [18, 19].

The primary inclusion criterion for the present study was the diagnosis of a DLSS according to the 10th revision of the International Classification of Diseases [20]. The diagnosis of DLSS was based on clinical evaluation by the treating spine surgeon as well as on MRI findings. Exclusion criteria were an age under 18, history of lumbar spine surgery, insufficient knowledge of the German language, severe cognitive impairment, and guardianship. The patients participated voluntarily without compensation. All participants signed an informed consent prior to participation. They completed the study questionnaires at the hospital or at home and then returned the questionnaires by mail. The study procedure was approved by the local ethics committee, RWTH Aachen University (IORG0006299), (EK026/15) and conducted according to the Declaration of Helsinki.

### Morphological markers

The morphological markers were evaluated with MRI of the lumbar spine. Two of the authors (VQ and YE) evaluated the MRI images; they were blinded to the clinical symptoms and radiological report. They classified severity of lumbar stenosis according to Schizas’ classification [21]: Grade A (mild), B (moderate), C (severe) or D (very severe). They documented the number of stenotic segments and presence of spondylolisthesis.

#### Patient reported outcomes

Patients completed the 10-item DESC-I, a Rasch-based depression screener (DESC) [22, 23]. It is a well validated screening instrument measuring depression severity on which patients describe symptom severity during the past 2 weeks using a rating scale ranging from 0 (never) to 4 (always). DESC-I scores range from 0 to 40 with higher scores indicating greater severity of depressive symptoms. A cut-off score of 12 corresponds to an interview-based diagnosis of a depressive episode according to ICD-10 criteria [24, 25].

Fear of movement and (re)injury was assessed using the 11-item German Version of the Tampa Scale for Kinesiophobia (TSK-GV) [26]. The TSK-GV is composed of the two subscales, TSK-somatic focus (TSK-SF, 5 items, score range: 5–20) and TSK-activity avoidance (TSK-AA, 6 items, score range: 6–24). While the TSK-SF subscale assess fear of injury in general, TSK-AA assesses avoidance of social and physical activities to minimize pain. Each item is rated on a 4-point rating scale (1 = “strongly disagree” to 4 = “strongly agree”) with higher scores indicating greater fear of movement and (re)injury.

ADLs were measured using a paper-pencil version of the Rasch-based RehaCAT-system [27]. RehaCAT assesses three aspects of ADL: (1) lower extremity functioning (RehaCAT-LE, e.g. walking, climbing stairs), (2) upper extremity (UE) functioning (RehaCAT-UE, e.g. self-care, eating) and (3) ADLs requiring both, upper and lower extremity functioning (RehaCAT-ADL, e.g. climbing stairs while carrying something heavy). Patients used a rating scale ranging from 0 (“without any difficulty”) to 4 (“impossible”) for all activities and indicated whether they were able to perform them without any help. RehaCAT-LE consists of 32 items (score range: 0–128), RehaCAT-UE of 37 items (score range: 0–148) and RehaCAT-ADL of 31 items (score range: 0–124). Higher scores indicate greater disability.

The Pain Interference Scale-German (PI-G) is a 28-item scale assessing the negative effects of pain on functioning [28]. It consists of three subscales assessing pain interference with mental functioning (PI-G-mental: cognition, emotion; 13 items, score range: 0–52), with recreational, household and work activities (PI-G-functional, 11 items, score range: 0–44) and with physical activities like walking or standing (PI-G-physical, 4 items, score range: 0–16). PI-G items assess how much pain interfered with daily life activities during the last 7 days using a 5-point rating scale ranging from 0 (“not at all”) to 4 (“very much”). Higher scores indicate greater pain interference.

The participation subscale of the Aachen Activity and Participation Index (AAPI-Part) [29] assessed the extent of participation in social, daily and work-related activities. AAPI-Part consists of 15 items which are rated on a 5-point rating scale (0 = “I couldn’t do it at all” to 4 = “without any problems”, score range: 0–60). Lower scores indicate lower participation levels.

### Statistical analyses

We calculated the prevalence of morphological markers (Schizas classification grades A, B, C & D), number of stenotic segments and presence of a spondylolisthesis (yes / no). Likewise, we calculated the percentage of patients with DESC-values above the clinical cut-point. We calculated mean scale sum scores, standard deviations as well as observed sum score ranges for patient-reported outcomes (PROs) to characterize the participants.

Prior to conducting linear regression analyses, we calculated correlations between the predictor variables and the dependent variables to estimate the relationship among these variables. The predictor variables for linear regression analyses were the sociodemographic variables age and gender, duration of disease, the three morphological markers (Schizas classification, number of stenotic segments and presence of a spondylolisthesis) as well as depression (DESC) and fear of movement (TSK subscales). The dependent variables were pain interference (PI-G subscales), the performance of ADLs (RehaCAT subscales) and participation (AAPI-Part). We calculated point biserial correlations for dichotomous variables (gender and presence of a spondylolisthesis) and Pearson’s correlation coefficients for continuously measured variables. Correlation coefficients (r) above |.5| were considered as evidence of strong correlations, coefficients between |.3| and |.5| as medium and coefficients between |.1| and |.3| as weak correlations.

Subsequently, stepwise linear regression analyses were conducted to predict interference of pain with the performance of ADLs (PI-G), ADLs (RehaCAT) and participation in social life (AAPI). We included only those predictor variables which were significantly correlated with the respective outcome variable. Multicollinearity among the predictor variables was controlled using the Variance Inflation Factor (VIF). Data analysis was performed using SPSS 24. Missing data were excluded list wise.

## Results

The characteristics of study participants are presented in Table 1. Study participants on average were 62.5 years old (SD = 11.7), and 50.7% were female.

**Table 1:** Sociodemographic characteristics of 67 patients with lumbar spine stenosis

Sociodemographic Characteristics	% of patients	Mean (SD)
Age		62.5 (11.7)
Gender		
female	50.7%	
male	49.3%	
Marital status		
married	59.1%	
single	9.1%	
separated/divorced	13.6%	
living with partner	6.1%	
widowed	12.1%	
Current work status		
employed for wages	34.8%	
retired	36.4%	
disability pension	9.1%	
unemployed	7.6%	
homemaker	10.6%	
partial pension	1.5%	

### Morphological markers

Table 2 shows the clinical characteristics of the sample. Most patients experienced chronic dorsal pain as the mean duration of the disease was 6.9 years (SD: 9.2; range: 0–37 years). In over 70% of the patients more than one segment was affected by stenosis. About one third of the patients exhibited an additional spondylolisthesis. According to the Schizas classification [21] about one third of the sample was classified as moderate (B), severe (C) or extreme (D).

**Table 2:** Clinical characteristics

Morphological Characteristics	% of patients
Spondylolisthesis	35.8%
Number of affected segments	
1	28.4%
2	41.8%
3	19.4%
4	10.4%
Schizas-classification	
B (moderate)	32.8%
C (severe)	35.8%
D (extreme)	31.3%
Depression (Patient Reported Outcome)	
DESC ≥12	41.5%)
Duration of disease	M(SD), Range
Duration of disease (years)	6.9 (9.2), 0–37

*DESC* Rasch-based depression screening

### Patient reported outcomes

Overall, patients reported a moderate to medium level of impairment with regard to pain interference and limitations in ADLs (see Table 3). The range of reported problems and limitations varied considerably between mild to severe limitations. Importantly, 41.5% of the patients had a depression score above the cut-point for depression indicating a high prevalence of clinically relevant depressive symptoms.

**Table 3:** Patient Reported Outcomes

Patient Reported Outcomes	Assessed construct	Possible score-range	M (SD)	Median	Range
Predictor variables					
DESC	Depression	0–40	10.9 (8.6)	9	0–35
TSK	Fear of movement:				
TSK_SF	Somatic focus	5–20	10.5 (3.3)	10.5	5–19
TSK_AA	Activity avoidance	6–24	14.4 (4.1)	14	6–23
Dependent variables					
PI-G	Pain interference:				
PI-G_mental	Mental	0–52	26.7 (10.6)	27	0–46
PI-G_functional	ADL	0–44	30.4 (11.1)	33	0–44
PI-G_physical	Mobility	0–16	10.9 (4)	12	0–16
RehaCAT	Activities of Daily Living:				
RehaCAT_LE	Lower extremity function	0–128	58.2 (29.8)	58.5	7–124
RehaCAT_UE	Upper extremity function	0–148	26.5 (29.2)	16	0–122
RehaCAT_ADL	ADL (UE & LE)	0–124	69 (27.6)	73	8–123
AAPI_P^a^	Participation	60–0	44 (12.2)	47	60–10

*DESC* Rasch-based depression screening, *TSK* Tampa Scale for Kinesiophobia, *SF* Somatic focus, *AA* Activity avoidance, *PI-G* Pain Interference – German, *LE* Lower extremity, *UE* Upper extremity, *ADL* Activities of daily living, *AAPI_P* Aachener Activity- and Participation Index, sub-scale participation; ^a^ *AAPI* only scale in which higher scores indicate better functioning

### Correlation analyses

Table 4 shows the pattern of correlations among the predictor and dependent variables. Among the morphological markers stenosis severity was correlated significantly with the degree of activity limitations in activities requiring lower extremity functioning (RehaCAT-LE); the correlation of .32 was of medium size. The number of stenotic segments was correlated negatively albeit weakly with pain interference (PI-G mental: r = −.28; PI-G functional: r = −.25) and positively but weakly with participation in daily activities (AAPI-Part; r = .26), indicating that patients with more affected segments reported less pain interference and more participation. Disease duration and performance of ADLs (RehaCAT_ADL) were correlated weakly (r = −.27). The association between the psychological predictors, depression (DESC) and fear of movement (TSK subscales) with all dependent variables were statistically significant with medium to strong effects.

**Table 4:** Correlations

	Gender	Age	Disease Duration	Spondylolisthesis	N. of segments	Schizas-Class.	DESC	TSK_SF	TSK_AA
PI-G_mental	.22	−.15	.05	.18	−.28*	−.16	.55**	.28*	.25*
PI-G_functional	.29*	−.19	−.07	−.01	−.25*	−.03	.48**	−12	.30*
PI-G_physical	.24	−.08	.04	−.11	−.04	.06	.35**	.26*	.21
RehaCAT_LE	.20	.11	−.17	−.14	−.03	.32*	.43**	.52**	.45**
RehaCAT_UE	−.03	.13	.02	−.01	−.04	.23	.40**	.64**	.45**
RehaCAT_ADL	.29*	−.06	−.27*	−.18	−.17	.10	.45**	.43**	.44**
AAPI_P	−.09	.20	.16	.01	.26*	−.03	−.57**	−.39**	−.25

*N*. Number, *Schizas-Class*. Schizas-Classification, *DESC* Rasch-based depression screening, *TSK* Tampa Scale for Kinesiophobia, *SF* Somatic focus, *AA* Activity avoidance, *PI-G* Pain Interference – German, *LE* Lower extremity, *UE* Upper extremity, *ADL* Activities of daily living, *AAPI_P* Aachener Activity- and Participation Index, sub-scale participation; * *p* **< .**05; ** *p* **< .**01

Investigating the correlation pattern among the predictor variables, depression was not significantly correlated with any of the morphological markers. The same applied for fear of movement with the exception that the activity avoidance subscale (TSK-AA) correlated significantly with the severity of stenosis (r = .32, *p* < .01) indicating that more severe stenosis was associated with more activity avoidance. Depression and fear of movement were correlated significantly (DESC – TSK-AA: r = .33, p < .01; DESC – TSK-SF: r = .40, p < .01), as were the two TSK-subscales (TSK-SF – TSK-AA: r = .54, p < .01).

### Multiple linear regression analyses

The results of the stepwise linear regression analyses are shown in Table 5. A significant proportion of variance of all dependent variables was accounted for by the predictor variables, ranging from 12% (R^2^ = .12; PI-physical) to 40% (R^2^ = .40; RehaCAT-LE; AAPI-P). Neither of the sociodemographic variables, age and gender, significantly contributed to the prediction of the dependent variables. Among the morphological markers only stenosis severity (Schizas-classification) contributed to the prediction of ADLs requiring LE functioning (RehaCAT-LE).

**Table 5:** Stepwise linear regression analyses

Outcome variable	Model	Predictor variables	R^2^	ΔR^2^	β^a^
PIG_mental	1	DESC	.32	.32**	.57**
PIG_functional	1	DESC	.23	.23**	.48**
	2	DESC	.28	.05*	.40**
		TSK_AA			.24*
PIG_physical	1	DESC	.12	.12**	.35**
RehaCAT_LE	1	TSK_SF	.28	.28**	.53**
	2	TSK_SF	.35	.07*	.50**
		Schizas-Class.			.26*
	3	TSK_SF	.39	.04*	.41**
		Schizas-Class.			.24*
		DESC			.23*
RehaCAT_UE	1	TSK_SF	.40	.40**	.63**
RehaCAT_ADL	1	TSK_AA	.28	.28**	.52**
	2	TSK_AA	.38	.10**	.40**
		DESC			.35**
AAPI_P	1	DESC	.33	.33**	−.57**
	2	DESC	.40	.07*	−.45**
		TSK_SF			−.29*

β^a^: please note that the standardized β coefficient is reported; *Schizas-Class*. Schizas-Classification, *DESC* Rasch-based depression screening, *TSK* Tampa Scale for Kinesiophobia, *SF* Somatic focus, *AA* Activity avoidance, *PI-G* Pain Interference – German, *LE* Lower extremity, *UE* Upper extremity, *ADL* Activities of daily living, *AAPI_P* Aachener Activity- and Participation Index, subscale participation; * *p* **< .**05; ** *p* **< .**01;

Depression and fear of movement were the most powerful predictors explaining the largest proportions of variance. Depression was the only significant predictor of pain interference with mental and physical functioning (PIG-mental; R^2^ change = .32, F(1, 58) = 27.5, *p* < .01; PIG-physical; R^2^ change = .12, F(1, 59) = 8.3, p < .01), and the strongest predictor of pain interference with the performance of recreational, household and work activities (PIG-functional; R^2^ change = .23, F(1, 59) = 17.5, *p* < .01) and of participation in social, daily and work-related activities (AAPI-P; R^2^ change = .33, F(1, 56) = 27, *p* < .01). Whereas the former was additionally predicted by the activity avoidance subscale of the TSK (PIG-functional; R^2^ change = .05, F(1, 58) = 4.31, *p* < .05), the prediction of the latter was improved significantly by adding the somatic focus-subscale of the TSK (AAPI-P; R^2^ change = .07, F(1, 55) = 6.5, p < .05). For the prediction of ADLs as assessed by the three RehaCAT-subscales, fear of movement was the strongest predictor. The somatic focus-subscale of the TSK was the only significant predictor of ADLs requiring UE functioning (RehaCAT_UE; R^2^ change = .40, F(1, 55) = 36.8, *p* < .01), and significantly predicted ADL requiring LE functioning (RehaCAT_LE; R^2^ change = .28, F(1, 59) = 22.9, p < .01) which was also predicted by the severity of stenosis (Schizas-classification; R^2^ change = .07, F(1, 58) = 6.07, p < .05) and depression (R^2^ change = .04, F(1, 57) = 4.13, p < .05). Activity avoidance and depression predicted the performance of ADLs requiring LE and UE at the same time (RehaCAT-ADL; TSK_AA: R^2^ change = .28, F(1, 48) = 18.2, p < .01; DESC: R^2^ change = .10, F(1, 47) = 7.9, p < .01).

## Discussion

The increasing number of affected patients and the growing socioeconomic consequences of DLSS underscore the need for an accurate diagnosis. Clinical judgement is generally based on symptom severity, functional deficits, physical examination and radiographic imaging [6]. However, psychological and sociodemographic factors as well as the course of disease, seem to influence the clinical presentation of DLSS (e.g. [13–15]). To understand better which factors influence the clinical presentation of DLSS, the present study investigated to what extent sociodemographic characteristics, morphological markers as well as depression and fear of movement predict limitations in ADLs and participation in social life in a sample of mostly chronic DLSS patients without a history of surgery. Results of the stepwise regression analyses revealed that pain interference with ADLs as well as limitations in ADLs and participation could be significantly predicted with the proportion of explained variance ranging between 12% (R^2^ = .12; PI-G-physical) and 40% (R^2^ = .40; RehaCAT-LE; AAPI-P). Neither the sociodemographic characteristics age and gender nor disease duration nor the morphological markers number of affected segments and the presence of spondylolisthesis contributed significantly to the prediction. Among the morphological markers only stenosis severity contributed to the prediction of ADLs requiring lower extremity functioning, revealing that more severe stenosis is associated with greater limitations. The variables contributing the most to the prediction of the dependent variables were the psychological factors depression and fear of movement. Depression was the strongest predictor for limitations in participation in social life as well as for pain interference with mental and physical functioning and with the execution of recreational and household activities. Fear of movement was the strongest predictor of limitations in ADLs.

The minor contribution of morphological markers in predicting ADL limitations is consistent with published findings. Most studies investigating the association between the severity of lumbar stenosis and clinical findings (e.g., pain interference, pain intensity, depression, walking distance, gait) did not report any strong correlations [8–10]. When significant correlations were found, they were generally of small size (e.g. [11], our study). Interestingly, the direction of the associations is inconsistent. In our study more severe stenosis was associated with greater limitations in ADLs requiring lower extremity functioning. Likewise, Hong et al. reported a significant, but small correlation between the number of affected segments (single vs. multilevel DLSS) and pain interference [11]. In contrast, Sigmundsson et al. reported better general health and less leg and back pain in patients with multilevel stenosis as compared to patients with single-level stenosis [9]. This finding is in agreement with our study. We found a significant, but small negative correlation between the number of stenotic segments and pain interference and a significant positive correlation with participation in daily activities. This finding suggests that under special circumstances patients with more affected segments, probably because of adaption, report less pain interference and more participation than patients with only a single affected segment. The results of Kuittinen et al.’s study point in a similar direction. They found a complex association between severity of DLSS and clinical findings, with partly milder symptoms in patients with more severe stenosis; they concluded that DLSS might not solely be an anatomical disorder, but that it has other underlying pathobiological mechanisms [10].

The literature spotlights psychological factors, as for example depression, as potential risk factors for poor clinical presentation (e.g., [13–15]). In our study a total of 41.5% of the patients had a clinically noticeable depression score. Furthermore, depression and fear of movement were the most powerful predictors of functional disability. The finding that fear of movement is an important predictor for limitations in ADLs and participation is in accordance with Lotzke et al.’s revised fear avoidance model (FAM) [30]. This model was developed to explain the progression of chronic musculoskeletal pain and disability [31]. According to this model, patients with a high pain-related fear, fear of movement and reduced self-efficacy are at risk of developing disabilities, depressed mood and low levels of physical activity [30]. Fear of movement as a protective reaction may be adaptive in the short term but may worsen the problem in the long term [31]. In accordance with Vlaeyen & Linton [31], Lotzke et al. suggested identifying and targeting fear of movement preoperatively to achieve a better postoperative functional outcome [30]. Evidence in support of FAM also comes from [32] who predicted short term pain and disability following lumbar disc surgery.

Interindividual differences in reacting to pain experiences might be responsible for the inconsistent findings regarding the association between morphological markers and clinical findings. Although being indispensable for the diagnosis, morphology seems to play a minor role in explaining limitations in ADLs. Here, the way patients are able to handle the experience of pain seems to be more important as proposed by the FAM [31]. A surgeon should be aware of this influence of psychological factors when choosing the optimal intervention with regard to prognosis on quality of life after treatment.

DLSS is one of the most frequent indications for spinal surgery [33] and in literature surgical therapy, particularly recommended for severe stenosis, seems to be superior to conservative treatments [34]. Many studies show that operative treatment is associated with greater improvement of pain and function in the first years [34, 35], even so the benefits diminish over time [35]. But some patients do not benefit from surgery. Fritsch et al. conducted a meta-analysis about the clinical course of pain and disability following surgery for spinal stenosis. They report, that in general patients experienced extensive reductions in pain and disability in the first 3-month post-surgery though pain level and disability persisted at 5 years follow up [36]. Further, surgery for spinal stenosis seems to affect depression 1 year later. However, persistence of depression after surgery correlated with a worse clinical outcome and a higher rate of unmet expectations [37, 38]. In a Cochrane database analysis, Zaina et al. found 10 to 24% complications in surgical treatment and no complications in non-operative treatment [39]. Repeated operations may result from recurrent spinal stenosis or an increasing spondylolisthesis [40]. In a metanalysis Machado et al. reported a reoperation rate from 3 to 28% [41].

Readers should note several study limitations. While the sample size allowed us to detect small effects, it was not sufficiently large to cross validate the results. We recruited patients during in- and outpatient therapy from two clinics; results may not generalize to other clinics and regions. Because patients were free to decline study participation, self-selection bias may limit generalizability. Nonetheless, findings are consistent with prior studies and support the need to screen for and treat psychological distress before surgery.

Altogether, it is difficult but extremely important for the surgeon to identify patient factors that contribute to the understanding of who is most likely to benefit from surgery. To do so, clinical information, morphological markers and psychological variables, in particular depression and fear of movement, must be taken into account. Future studies should investigate whether psychological screening or a preoperative psychological consultation helps avoid operations or achieves better postsurgical outcomes.

## Conclusions

Psychological factors may be a risk factor for a worse clinical presentation and a poorer postoperative outcome. Early psychological screening and treatment, when needed, is recommended and should be implemented routinely. This protocol may help avoid operations with a low likelihood of functional improvement and, direct treatment to patients with psychological disease who may achieve better postoperative outcomes with treatment. Further studies must show to what extent psychological screening and treatment enables better patient outcomes.

## Data Availability

The datasets used and/ or analyzed during the current study are available from the corresponding author on reasonable request.

## Abbreviations

AAPI_P: Aachener Activity- and Participation Index, sub-scale participation
ADL: Activities of daily living
DESC: Rasch-based depression screening
DLSS: Degenerative lumbar spinal stenosis
LE: Lower extremity
MRI: Magnetic resonance imaging
N: Number
PI-G: Pain Interference – German
PROs: Patient-reported outcomes
r: Correlation coefficient
Schizas-Class.: Schizas-Classification
SD: Standard deviation
TSK-AA: Tampa Scale for Kinesiophobia, subscale activity avoidance
TSK-GV: Tampa Scale for Kinesiophobia
TSK-SF: Tampa Scale for Kinesiophobia, sub-scale somatic focus
UE: Upper extremity
VIF: Variance Inflation Factor
